# Histone Deacetylase 2 Is a Component of Influenza A Virus-Induced Host Antiviral Response

**DOI:** 10.3389/fmicb.2017.01315

**Published:** 2017-07-17

**Authors:** Prashanth T. Nagesh, Mazhar Hussain, Henry D. Galvin, Matloob Husain

**Affiliations:** ^1^Department of Microbiology and Immunology, University of Otago Dunedin, New Zealand; ^2^Department of Microbiology, New York University School of Medicine, New York NY, United States

**Keywords:** influenza A virus, histone deacetylase 2, HDAC2, HDAC1, proteasomal degradation, host antiviral response, viperin, STAT1

## Abstract

Host cells produce variety of antiviral factors that create an antiviral state and target various stages of influenza A virus (IAV) life cycle to inhibit infection. However, IAV has evolved various strategies to antagonize those antiviral factors. Recently, we reported that a member of class I host histone deacetylases (HDACs), HDAC1 possesses an anti-IAV function. Herein, we provide evidence that HDAC2, another class I member and closely related to HDAC1 in structure and function, also possesses anti-IAV properties. In turn, IAV, like HDAC1, dysregulates HDAC2, mainly at the polypeptide level through proteasomal degradation to potentially minimize its antiviral effect. We found that IAV downregulated the HDAC2 polypeptide level in A549 cells in an H1N1 strain-independent manner by up to 47%, which was recovered to almost 100% level in the presence of proteasome-inhibitor MG132. A further knockdown in HDAC2 expression by up to 90% via RNA interference augmented the growth kinetics of IAV in A549 cells by more than four-fold after 24 h of infection. Furthermore, the knockdown of HDAC2 expression decreased the IAV-induced phosphorylation of the transcription factor, Signal Transducer and Activator of Transcription I (STAT1) and the expression of interferon-stimulated gene, viperin in infected cells by 41 and 53%, respectively. The role of HDAC2 in viperin expression was analogous to that of HDAC1, but it was not in the phosphorylation of STAT1. This indicated that, like HDAC1, HDAC2 is a component of IAV-induced host innate antiviral response and performs both redundant and non-redundant functions vis-a-vis HDAC1; however, IAV dysregulates them both in a redundant manner.

## Introduction

Influenza A virus (IAV) is among the successful and unpredictable human pathogens, which have a global presence. IAV exhibits a broad host range and is known to infect humans, seals, horses, pigs, dogs, cats, and birds. In addition, IAV has also been detected in bats (Wu et al., 2014). The interspecies transmission of IAV regularly occurs and is especially common among birds, between humans and pigs, and pigs to poultry (Hussain et al., 2017). This gives rise to new IAV strains that are the cause of regular seasonal epidemics and unpredictable pandemics, and lately, recurrent and often fatal zoonotic outbreaks in humans (Husain, 2014). In humans, IAV is transmitted via aerosol and causes an acute febrile respiratory disease, which is commonly known as ‘flu.’ Flu is particularly severe in the children ≤ 5 years old, adults ≥ 65 years old, and at-risk individuals. Influenza viruses continue to present a significant burden on global public health and economy (Nair et al., 2011; Gasparini et al., 2012; Peasah et al., 2013). The World Health Organization (WHO) estimates that, worldwide, influenza virus causes approximately 1 billion flu cases, 3–5 million cases of severe illness, and 300,000 to 500,000 deaths, annually. A universal influenza vaccine has not been developed yet though influenza type- and subtype-specific vaccine can be developed. The alternating annual influenza vaccination program comprising the reformulated vaccine is the key measure to prevent or control seasonal influenza epidemics in Northern and Southern Hemispheres. In addition, adamantanes and neuraminidase inhibitors are two classes of antiviral drugs that have been approved for treatment of influenza virus infections. However, influenza virus resistance to these drugs emerges rapidly and is a significant concern. Consequently, neuraminidase-inhibitors are the only class of drugs that are currently in use, because vast majority of circulating influenza subtypes are resistant to adamantanes (Dong et al., 2015; Hussain et al., 2017). Furthermore, several circulating influenza viruses have also acquired mutations conferring the resistance to neuraminidase inhibitors (McKimm-Breschkin, 2013; Hussain et al., 2017). Therefore, there is an urgent need of novel, alternative, effective, and long-lasting anti-influenza intervention strategies. One strategy is to identify novel anti-influenza host factors and understand their interplay with influenza virus, and subsequently target them to strengthen host defenses against influenza. Host cells produce variety of factors that create an antiviral state and target various stages of virus life cycle to prevent infection (Schneider et al., 2014); however, viruses have evolved various strategies to antagonize those antiviral factors (Duggal and Emerman, 2012). Recently, we and others have discovered that host histone deacetylases (HDACs) have anti-IAV function (Husain and Cheung, 2014; Koyuncu et al., 2014; Nagesh and Husain, 2016).

The HDACs are a family of host enzymes that catalyze the deacetylation of acetylated proteins. Acetylation is a post-translational modification of proteins that is known to occur in a variety of nuclear and cytoplasmic proteins (Choudhary et al., 2009). Acetylation and deacetylation is a reversible process controlled by histone acetyltransferases (HATs) and HDACs, respectively, and influences diverse host cell processes, for example cell cycle, gene expression, signaling, and innate immune response (Shakespear et al., 2011; Benedetti et al., 2015; Haery et al., 2015). Consequently, HDACs has been exploited as a target for treatment of various human diseases, especially cancer (Benedetti et al., 2015). HDACs are ubiquitously expressed in all eukaryotic cells. The family of mammalian HDACs is comprised of at least 18 members, which are classified into four classes. The classes I, II, and IV HDACs are known as classical HDACs and are zinc-dependent; whereas, the class III HDACs are known as sirtuins and require NAD^+^ for enzymatic activity (Benedetti et al., 2015; Mei et al., 2016). The class I is comprised of four members namely HDAC 1, 2, 3, and 8. The class II has been sub-divided into classes IIa and IIb, with four members (HDAC 4, 5, 7, and 9) and two members (HDAC 6 and 10), respectively. The class III is comprised of seven members namely sirtuin (SIRT) 1 to 7. Finally, only one member, HDAC11 has been assigned to class IV that shares homology to both class I and class II HDACs. The HDACs in each class differ in their structure, enzymatic activity, intracellular distribution, and expression profile in different tissues (Benedetti et al., 2015; Mei et al., 2016).

In addition to human diseases, HDACs are also involved in the viral infections, albeit in a paradoxical manner (Herbein and Wendling, 2010; Budayeva et al., 2015). During the latent infection, HDACs promote viral latency, but during the acute infection, HDACs inhibit viral multiplication. We discovered that both class I and class II HDACs possess anti-IAV activity (Husain and Harrod, 2011), and at least one member of class I (HDAC1) and class II (HDAC6) specifically inhibits IAV infection (Husain and Cheung, 2014; Nagesh and Husain, 2016). Further, Koyuncu et al. (2014) reported that almost all seven sirtuins possess anti-IAV properties. In addition, HDAC 1, 2, 6 and some sirtuins also exhibit antiviral function against other human viruses such as gammaherpesvirus, human immunodeficiency virus-1, herpes simplex virus-1, cytomegalovirus and adenovirus, and animal viruses such as porcine reproductive and respiratory syndrome virus (Valenzuela-Fernandez et al., 2005; Koyuncu et al., 2014; Mounce et al., 2014; Lu et al., 2017). This indicates an emerging broad antiviral spectrum of the host HDACs and opportunity to exploit them for developing alternative antiviral strategies. Therefore, it is imperative to identify the anti-IAV potential of individual HDACs and elucidate their antiviral mechanisms. An antiviral role for HDAC2 during IAV infection has not been demonstrated. We provide evidence herein that, like other class I member HDAC1, HDAC2 also exhibits anti-IAV properties and is an important component of host innate antiviral response against IAV.

## Materials and Methods

### Cells and Virus Strains

A549 and MDCK cells (Husain and Cheung, 2014; Nagesh and Husain, 2016) were grown and maintained in minimum essential medium (MEM; Life Technologies) supplemented with 10% fetal bovine serum (FBS; Sigma–Aldrich), penicillin-streptomycin, and L-glutamine (Life Technologies) at 37°C and under 5% CO_2_ atmosphere. Influenza virus A/PR/8/34 (H1N1), A/New Caledonia/20/1999 (H1N1), and A/WSN/34 (H1N1) strains (Husain and Cheung, 2014; Nagesh and Husain, 2016), were propagated in 10-days old embryonated chicken eggs and titrated on MDCK cells.

### Infection

The cell monolayers were washed twice with serum-free MEM and the virus inoculum prepared in serum-free MEM was added to the cells, which were then incubated at 35°C for 1 h. After removing the virus inoculum, cells were washed once with serum-free MEM. Finally, fresh serum-free MEM was added to the cells and cells were incubated back at 35°C. In some experiments, NH_4_Cl (Sigma–Aldrich), MG132 (Calbiochem) or caspase 3 inhibitor (Calbiochem) were included in serum-free MEM. To inactivate IAV, virus inoculum was irradiated under 30W UV bulb for 5 min before adding to the cells. Generally, cells were infected with IAV at the multiplicity of infection (MOI) of 0.5 to 5.0 plaque-forming units (PFUs) per cell.

### Quantitative Real-Time PCR

The total RNA was extracted from cells using PureLink RNA isolation kit (Life Technologies). The RNA integrity was confirmed on Bioanalyzer 2100 (Agilent) using RNA 6000 Nano Chip and purity and quantity were measured on NanoDrop 1000 (ThermoFisher). The SuperScript III First-Strand Synthesis System (Life Technologies) and total RNA template was used to synthesize the cDNA. The quantitative real-time PCR was performed on ViiA 7 Real-Time PCR System (Applied Biosystems) by using the cDNA, SYBR green select master mix (Life Technologies) and pre-designed KiCqStart primers (Sigma–Aldrich). Primer sequence – HDAC2 forward: 5′-GGTCATGCTAATGTGTAGAAG-3′, HDAC2 reverse: 5′-GTCGGTCCAAAATACTCAAG-3′, 18S RNA forward: 5′-ATCGGGGATTGCAATTATTC-3′, 18S RNA reverse: 5′-CTCACTAAACCATCCAATCG-3′, GAPDH forward: 5′-CTTTTGCGTCGCCAG-3′, GAPDH reverse: 5′-TTGATGGCAACAATATCCAC-3′, beta-actin forward: 5′-GACGACATGGAGAAAATCTG-3′, beta-actin reverse: 5′-ATGATCTGGGTCATCTTCTC-3′, viperin forward: 5′-CTTTTGCTGGGAAGCTCTTG-3′, viperin reverse: 5′-CAGCTGCTGCTTTTCTCCTCT-3′. Beta-actin, 18S RNA, and GAPDH were used as reference genes for normalizing and determining the fold- or percent change in the expression of HDAC2 and viperin mRNAs, which was calculated using 2(-Delta Delta C(T)) method as described elsewhere (Livak and Schmittgen, 2001).

### Western Blotting

The cells were harvested and lysed in lysis buffer (50 mM Tris-HCl [pH 7.4], 150 mM NaCl, 0.5% sodium dodecyl sulfate [SDS], 0.5% sodium deoxycholate, 1% TritonX-100, and 1X protease-inhibitor cocktail [Roche]), and total protein was estimated using BCA kit (ThermoFisher). Equal amount of proteins were resolved on 10 or 15% Tris-glycine SDS-PAGE under reducing conditions. Proteins were transferred onto Protran Premium nitrocellulose membrane (GE Healthcare), which was then probed with either mouse anti-HDAC2 (clone 3F3) (1:1,000, Cell Signaling), rabbit anti-viperin (1:1,000; Clone D5T2X, Cell Signaling), mouse anti-STAT1 (1:1,000; Clone 42/Stat1, BD biosciences), mouse anti-STAT1 (pY701) (1:1,000; Clone, 14/P-STAT1, BD biosciences), mouse anti-ubiquitin (1:500; Clone, P4D1, Santa Cruz), mouse anti-NP (1:1,000; NR-4282, obtained through BEI Resources, NIAID, NIH), goat anti-NP (1:1,000; kindly provided by St Jude Children’s Research Hospital), rabbit anti-actin (1:5,000; Abcam) or rabbit anti-protein disulfide isomerase (PDI) (1:5,000; Sigma–Aldrich) antibody followed by IRDye 680LT- or IRDye 800LT-conjugated (1:10,000; Li-COR) or horseradish peroxidase-conjugated anti-mouse, anti-goat, or anti-rabbit IgG antibody (1:2,000–5,000; Life Technologies). Protein bands were visualized by either fluorescence or using a chemiluminescent ECL substrate (GE Healthcare). Images were acquired on Odyssey Fc imaging system (Li-COR) and exported as TIFF files, which were then cropped and compiled in Adobe Photoshop CC 2015.

### Knockdown of HDAC2 Expression

Pre-designed small-interfering RNA (siRNA) targeting HDAC2 (ID: SASI_Hs02_00332058) and non-targeting MISSION^®^ universal negative control obtained from Sigma–Aldrich were delivered to cells using Lipofectamine RNAiMax reagent (Life Technologies) by following the manufacturer’s guidelines. Briefly, siRNA oligonucleotides and RNAiMax were separately diluted in OptiMEM I medium (Life Technologies), then mixed together and incubated for 20–30 min at room temperature. Subsequently, the siRNA-RNAiMax complex was mixed with the cells and transferred to a cell culture plate. Cells were incubated at 37°C for 72 h before infecting or further processing them.

### Cell Viability Assay

The viability of cells was ascertained by performing the MTT (3-[4, 5-dimethylthiazol-2-yl]-2, 5 diphenyl tetrazolium bromide) assay (van Meerloo et al., 2011). Cells transfected with siRNA were washed twice with the phosphate-buffered saline (PBS), then treated with 1 ml MTT reagent (Sigma–Aldrich) and incubated at 37°C for 1 h. Subsequently, 1 ml dimethyl sulfoxide (Calbiochem) was added to the cells, which were further incubated for 15–20 min at room temperature with shaking. Finally, the absorbance was measured at 595 nm on Multiskan Ascent plate reader (LabSystems).

### Microplaque Assay

The culture medium harvested from infected cells was cleared off of cell-debris by low-speed centrifugation, supplemented with 0.3% BSA (Sigma–Aldrich) and titrated on MDCK cells to perform the microplaque assay. For this, confluent MDCK cell monolayers were infected with the 10-fold serial dilutions of culture medium as above. After removing the virus inoculum, cells were overlaid with serum-free MEM containing 1 μg/ml TPCK-trypsin (Sigma–Aldrich) and 0.8% Avicel (RC-581; FMC Biopolymer). The overlay was removed after 18–20 h incubation and cells were fixed with 4% formalin (Sigma–Aldrich) and subsequently permeabilized with 0.5% TritonX-100 in 20 mM glycine solution. Cells were then stained with mouse anti-NP antibody (1:1,000) followed by horseradish peroxidase-conjugated anti-mouse IgG antibody (1:1,000). The plaques were visualized by adding the True blue substrate (KPL biosciences).

### Statistical Analysis

The statistical analyses were performed using Prism 6 (GraphPad). The *P*-values were calculated using unpaired *t*-tests for pairwise data comparisons and one-way analysis of variance (ANOVA) or two-way ANOVA for multiple data set comparisons. A *P*-value of ≤ 0.05 was considered significant.

## Results

### HDAC2 mRNA Level is Downregulated in IAV-Infected Cells

**FIGURE 1:**
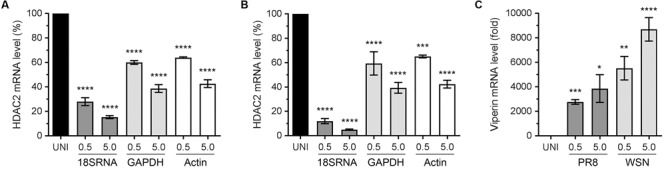
Influenza A virus (IAV) downregulates HDAC2 mRNA level. A549 cells were infected with PR8 **(A)** or WSN **(B)** at an MOI of 0.5 and 5.0 for 24 h. The uninfected (UNI) and infected (0.5, 5.0) cells were harvested, processed, and HDAC2 and viperin mRNAs were detected by quantitative real-time PCR. Alongside, the 18S RNA, GAPDH and actin mRNAs were detected as a reference and used to normalize the levels of HDAC2 **(A,B)** and viperin **(C)** mRNA. The normalized value of HDAC2 or viperin mRNA in UNI sample was considered 100% or one-fold, respectively, for comparison to infected sample. Error bar represents means ± standard errors of the means of three independent experiments; asterisks indicate the significant differences in means.

Recently, we described an antiviral role of host HDAC1 during IAV infection (Nagesh and Husain, 2016). The HDAC1 and HDAC2 are very similar proteins and share almost 82% amino acid identity as well as have some common domains and motifs (Brunmeir et al., 2009). It is believed that HDAC1 and HDAC2 originated from a common ancestor through a gene duplication event. Although HDAC1 and HDAC2 acquired some non-redundant and distinct biological functions during their evolution, they do retain some functional redundancy and complementarity (Brunmeir et al., 2009). Hence, we hypothesized that, like HDAC1, host HDAC2 also has an anti-IAV function and IAV dysregulates HDAC2 to undermine its antiviral function. To test this hypothesis, we first determined the HDAC2 transcript level in response to the IAV infection. Human lung epithelial A549 cells were infected with the influenza virus A/PR/8/34(H1N1) strain (hereafter referred to as PR8) or A/WSN/34(H1N1) strain (hereafter referred to as WSN) at an MOI of 0.5 and 5.0. After 24 h of infection, cells were processed to measure the HDAC2 mRNA level by quantitative real-time PCR (qPCR). In addition, the mRNA levels of three reference genes – 18S ribosomal RNA (18S RNA), glyceraldehyde 3-phosphate dehydrogenase (GAPDH), and actin were measured as the normalizing controls. Furthermore, the mRNA level of interferon-stimulated gene (ISG), viperin was measured as a positive control for infection as well as qPCR. We found that both PR8 (**Figure 1A**) and WSN (**Figure 1B**) downregulated the HDAC2 mRNA level in A549 cells in a statistically significant and dose-dependent manner. However, the extent of HDAC2 mRNA downregulation was dependent on the reference gene used for normalization. We found that when normalized to GAPDH, the infection of A549 cells with PR8 at an MOI of 0.5 and 5.0 caused a significant 40% (*P* = 0.0001) and 61.3% (*P* = 0.0001) reduction in HDAC2 mRNA level, respectively (**Figure 1A**). Likewise, when normalized to GAPDH, the infection with WSN at an MOI of 0.5 and 5.0 caused a significant 40.6% (*P* = 0.0001) and 60.6% (*P* = 0.0001) reduction in HDAC2 mRNA level, respectively (**Figure 1B**). Similarly, when normalized to actin, the HDAC2 mRNA levels in A549 cells were found to be reduced by a significant 36% (*P* = 0.0001) and 57.3% (*P* = 0.0001) when infected with the PR8 at an MOI of 0.5 and 5.0, respectively (**Figure 1A**), and a significant 35% (*P* = 0.0003) and 57.6% (*P* = 0.0001) when infected with the WSN at an MOI of 0.5 and 5.0, respectively (**Figure 1B**). However, when normalized to 18S RNA, the reduction in HDAC2 mRNA levels in A549 cells was found to be 72% (*P* = 0.0001) and 84.6% (*P* = 0.0001) after infection with PR8 at an MOI of 0.5 and 5.0, respectively (**Figure 1A**), and a profound 88% (*P* = 0.0001) and 95% (*P* = 0.0001) after infection with WSN at an MOI of 0.5 and 5.0, respectively (**Figure 1B**). The expression level of viperin is known to increase in response to virus infection (Fitzgerald, 2011). Consistent with this, there was a significant and dose-dependent increase in viperin mRNA levels in response to infection with both PR8 and WSN when normalized to 18S RNA (**Figure 1C**) as well as GAPDH and actin (Supplementary Figure 1). These data indicated that IAV downregulates HDAC2 mRNA level in H1N1 strain-independent manner.

### HDAC2 Polypeptide Level is Downregulated in IAV-Infected Cells

**FIGURE 2:**
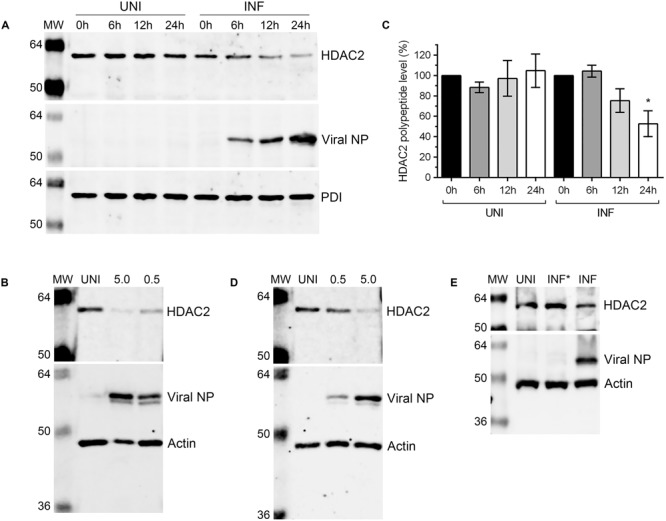
Influenza A virus downregulates HDAC2 polypeptide level. **(A)** A549 cells were infected with WSN at an MOI of 0.5 and harvested at the indicated times. Total cell lysates were prepared, and HDAC2 (60 kDa), viral NP (56 kDa), and PDI (57 kDa) were detected in uninfected (UNI) and infected (INF) cell lysates by WB. The PDI was detected as a loading control and NP was detected as an infection marker. **(B)** The HDAC2 and PDI protein bands were quantified using Image Studio Lite V5.0 software (Li-COR) and the amount of HDAC2 was normalized to corresponding PDI amount. The normalized amount of HDAC2 at 0 h in UNI and INF samples was considered 100% for comparisons to the respective 6, 12, and 24 h samples. Data presented are means ± standard errors of the means of three independent experiments; asterisks indicate the significant differences in means. A549 cells were infected with IAV PR8 **(C)** or New Caledonia **(D)** strains at an MOI of 0.5 or 5.0, or with live (INF) or UV-irradiated (INF^∗^) WSN at an MOI of 0.5 **(E)** for 24 h. The HDAC2, NP and actin (42 kDa; as a loading control) were detected in total cell lysates by WB. MW, molecular weights.

After detecting a significant reduction in HDAC2 mRNA level in IAV-infected cells, we next sought to determine whether HDAC2 polypeptide level is also downregulated in IAV-infected cells. A549 cells were infected with WSN at an MOI of 0.5, and the infected and corresponding uninfected cells were harvested after 0, 6, 12, and 24 h of infection (‘0 h’ is the time point when virus inoculum is removed after 1 h incubation – see Materials and Methods). The total cell lysates were prepared and equal protein amounts were resolved on SDS-PAGE to detect the HDAC2, viral nucleoprotein (NP), and PDI polypeptides by western blotting (WB). The viral NP was detected as an infection marker and the PDI was detected as a control. We found that the level of HDAC2 polypeptide (∼60 kDa) in WSN-infected cells decreased with the progress of infection, peaking at 24 h; whereas, it remained largely unchanged throughout in uninfected cells (**Figure 2A**). To quantitate this decrease in HDAC2 polypeptide level, the intensity of all HDAC2 and PDI bands was quantified using the Image Studio Lite software (Version 5.0, LI-COR), and the amounts of HDAC2 was normalized with the corresponding PDI amounts. Subsequently, the amounts of HDAC2 at 0 h in uninfected and infected cells were considered 100% to compare its amount in respective samples at subsequent time points. We found that there was a statistically non-significant 24.7% and a significant 47.3% (*P* = 0.02) decrease in HDAC2 polypeptide levels in infected cells after 12 and 24 h of infection, respectively (**Figure 2B**). However, in uninfected cells, there was no significant change in the HDAC2 polypeptide levels between 0 and 24 h (**Figure 2B**). To determine whether, like mRNA, the reduction in HDAC2 polypeptide level was independent of IAV strain, A549 cells were infected with PR8 at an MOI of 0.5 or 5.0. Cells were then harvested after 24 h and processed to detect HDAC2, NP and actin polypeptides by WB. Here, actin was detected as a control. We found that, like WSN, infection with PR8 also reduced the HDAC2 polypeptide level in A549 cells, and in a dose-dependent manner (**Figure 2C**). Both PR8 and WSN are considered as the prototypic IAV lab strains and have been used extensively to study IAV biology in the host (Coombs et al., 2010; Tse et al., 2013). Furthermore, because of its pathogenic nature, WSN is also considered by some as a model for highly pathogenic influenza viruses (Tse et al., 2013). Nevertheless, we next infected A549 cells with influenza virus A/New Caledonia/20/1999(H1N1) strain – a more clinically relevant IAV strain (Hartmann et al., 2015), at an MOI of 0.5 or 5.0 for 24 h to assess the downregulation of HDAC2 polypeptide. Consistent with the results obtained with WSN and PR8, New Caledonia strain also reduced the level of HDAC2 polypeptide in A549 cells in a dose-dependent manner (**Figure 2D**). Finally, we infected A549 cells with the UV-irradiated WSN inoculum (0.5 MOI) to confirm that a replication-competent IAV was needed to downregulate the HDAC2 polypeptide levels (**Figure 2E**).

### Downregulation of HDAC2 Polypeptide in Infected Cells Occurs via Proteasomal Degradation

**FIGURE 3:**
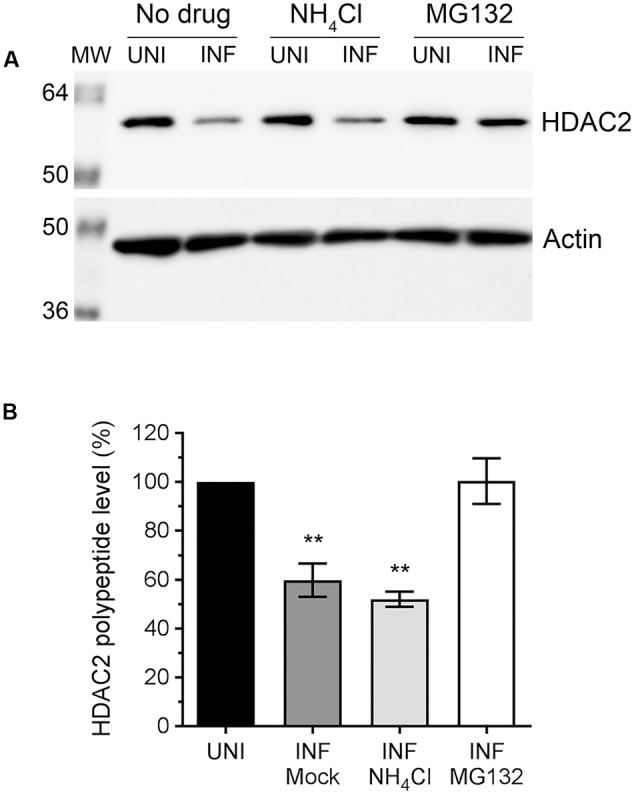
The downregulation of HDAC2 polypeptide occurs by proteasomal degradation. **(A)** A549 cells were infected with WSN at an MOI of 0.5, and subsequently treated with NH_4_Cl (20 mM) or MG132 (10 μM) for 24 h. Total cell lysates were prepared, and HDAC2 and actin were detected in uninfected (UNI) and infected (INF) cell lysates by WB. **(B)** The HDAC2 and actin protein bands were quantified as in **Figure 2B** and the amount of HDAC2 was normalized to corresponding actin amount. The normalized amount of HDAC2 in respective UNI samples was considered 100% for comparisons to INF samples. Data presented are means ± standard errors of the means of three independent experiments; asterisks indicate the significant differences in means. MW, molecular weights.

Above data indicated that IAV downregulates host HDAC2 expression, which potentially occurs mainly at the polypeptide level. Because, unlike mRNA levels, the HDAC2 polypeptide levels were reduced by all three IAV strains used in a similar manner. This prompted us to investigate whether IAV downregulates HDAC2 polypeptide level by promoting its degradation in infected cells. Primarily, two pathways, one governed by the proteasome and the other by lysosome, mediate the degradation of eukaryotic proteins. To identify the pathway employed by IAV to promote the degradation of HDAC2 polypeptide in infected cells, A549 cells were infected with WSN as above and treated with proteasome-inhibitor MG132 or lysosome-inhibitor NH_4_Cl using previously reported and tested concentrations of 10 μM and 20 mM, respectively (Wang et al., 2014; Nagesh and Husain, 2016). The levels of HDAC2 polypeptide were then analyzed, quantified and normalized with the corresponding actin levels as described above. We found that MG132 treatment rescued the downregulation of HDAC2 polypeptide in infected cells almost to the level of uninfected cells (**Figure 3A**). However, NH_4_Cl treatment did not have any effect on the downregulation of HDAC2 polypeptide in infected cells (**Figure 3A**). Compared to the corresponding uninfected cells, there was a significant 40.2% (*P* = 0.003) and 48% (*P* = 0.001) decrease in HDAC2 polypeptide levels in untreated and NH_4_Cl-treated infected cells, respectively (**Figure 3B**), which was consistent with the data presented in **Figure 2** above. In contrast, there was basically no decrease in the level of HDAC2 polypeptide in MG132-treated infected cells compared to the corresponding uninfected cells (**Figure 3B**). An increase in the level of ubiquitinated proteins detected in MG132-treated cells (Supplementary Figure 2B) and a reduced level of NP cleavage product (Supplementary Figure 2A, asterisks) detected in NH_4_Cl-treated infected cells confirmed the potency of MG132 and NH_4_Cl, respectively. The NP is known to be cleaved by caspases (Zhirnov et al., 1999), activation of which is known to be induced and inhibited by the lysosomes and NH_4_Cl, respectively (Appelqvist et al., 2013). Furthermore, the treatment with caspase 3 inhibitor also did not rescue the HDAC2 polypeptide level in infected cells (Supplementary Figure 3). Therefore, these data indicated that IAV promotes the degradation of HDAC2 polypeptide via host proteasomal pathway.

### IAV Grows to Higher Titers in HDAC2-Depleted Cells

**FIGURE 4:**
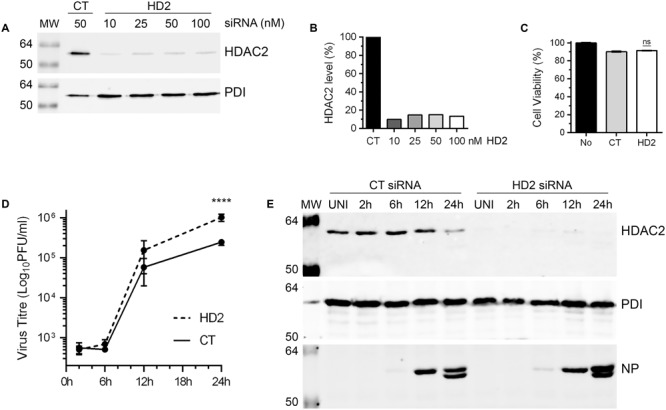
Influenza A virus grows to higher titers in HDAC2-depleted cells. **(A)** A549 cells were transfected with indicated concentrations of non-targeting control (CT) or HDAC2-targeting (HD2) siRNAs for 72 h. Total cell lysates were prepared and HDAC2 and PDI were detected by WB. **(B)** The HDAC2 and PDI bands were quantified as in **Figure 2B** and the amount of HDAC2 was normalized to corresponding PDI amount. The normalized amount of HDAC2 in control siRNA sample was considered 100% for comparisons to HDAC2 siRNA samples. **(C)** A549 cells were transfected with either no siRNA or 10 nM of the CT or HD2 siRNAs. After 72 h, the cell viability was determined by MTT assay; ns, not significant. **(D,E)** A549 cells were transfected with 10 nM of CT siRNA or HD2 siRNA for 72 h. Cells were then infected with WSN at an MOI of 0.5, and the culture medium and the cells were harvested separately after 2, 6, 12, and 24 h of infection. **(D)** The culture media was then titrated by microplaque assay to determine the yield of released infectious progeny. Data presented are means ± standard errors of the means of three independent experiments; asterisks indicate the significant differences in means. **(E)** The cells were used to prepare the total cell lysates, and detect HDAC2, PDI and NP polypeptides by WB. UNI, uninfected; MW, molecular weights.

Above data demonstrated that IAV downregulates the level of HDAC2 polypeptide in infected cells by at least 40%. This indicated a potential anti-IAV role for HDAC2 and by downregulating its expression, IAV is potentially minimizing the antiviral effect of HDAC2. If this is true, then IAV should grow to higher titers in the cells that are further depleted with HDAC2 expression. To assess this, IAV growth kinetics was analyzed in A549 cells with depleted expression of HDAC2, and RNA interference was employed to achieve this. First, to obtain a significant knockdown of HDAC2 expression, 10, 25, 50, and 100 nM of a siRNA targeting human HDAC2 and 50 nM of a non-targeting control siRNA were delivered to A549 cells, and subsequently the knockdown of HDAC2 expression was analyzed by WB. A significant knockdown of HDAC2 expression was achieved with all siRNA concentrations used (**Figure 4A**). However, the 10 nM concentration was chosen for subsequent experiments as it was adequate to knockdown the HDAC2 expression by 90% (**Figure 4B**), and with no cytotoxicity compared to the control siRNA (**Figure 4C**). Next, A549 cells transfected with either non-targeting control siRNA or HDAC2-targeting siRNA were infected with WSN at an MOI of 0.5, and the culture medium and the infected cells were separately harvested after 2, 6, 12, and 24 h of infection. The cells were processed and subjected to WB to confirm the knockdown of HDAC2 expression; whereas, the culture media were titrated by microplaque assay to measure the amount of released infectious IAV progeny. Indeed, we found that IAV grows to higher titers and exhibits a faster growth characteristic in HDAC2-depleted cells (**Figure 4D**). After 12 and 24 h of infection, the cells with depleted expression of HDAC2 released a statistically non-significant 2.6-fold and a significant 4.2-fold (*P* < 0.0001) more infectious progeny, respectively, than the control cells at the corresponding times (**Figure 4D**). The WB analysis of cells confirmed the significant knockdown of HDAC2 expression at each time point (**Figure 4E**). In addition, the downregulation of HDAC2 polypeptide in control siRNA-transfected cells after 24 h of infection was consistent with the data presented in **Figures 2, 3**. Furthermore, a significant 1.9-fold (*P* = 0.02) increase in intracellular NP level in HDAC2-depleted cells compared to control cells was also observed after 24 h infection by WB (**Figure 4E**). A fluorescence microscopy experiment was carried out to determine whether such increase was due to an increase in the NP level per cell or overall increase in the number of infected cells. The data obtained seem to indicate the latter (Supplementary Figure 4); however, further investigations involving the single-cell analyses are needed to ascertain this.

### HDAC2 Is Involved in IAV-Induced Host STAT1 Phosphorylation and Viperin Expression

**FIGURE 5:**
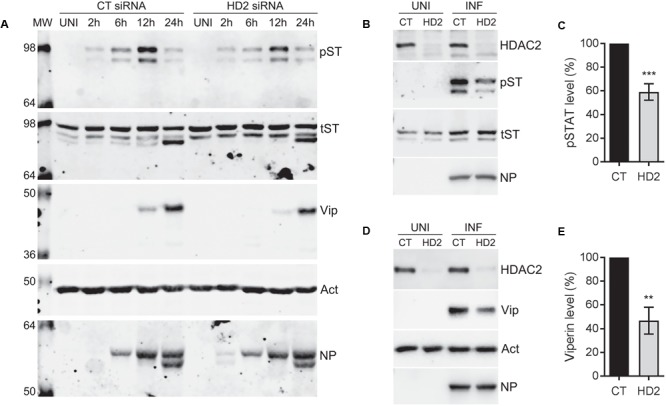
HDAC2 is involved in IAV-induced phosphorylation of STAT1 and expression of viperin. **(A)** A549 cells were transfected with non-targeting control (CT) siRNA or HDAC2-targeting (HD2) siRNA for 72 h, and then infected with the WSN at an MOI of 0.5. Cells were then harvested at the indicated times, total cell lysates were prepared, and phosphorylated STAT1 (pST, 91/84 kDa), total STAT1 (tST, 91/84 kDa), viperin (Vip, 42 kDa), and actin (Act) were detected in total uninfected (UNI) and infected (INF; 2, 6, 12, 24 h) cell lysates by WB. **(B,C)** A549 cells transfected with the control and HDAC2 siRNAs and infected with the WSN as above were harvested after 12 h of infection. **(B)** The HDAC2, phosphorylated STAT1, total STAT1 and NP were detected in total uninfected and infected cell lysates by WB. **(C)** The phosphorylated STAT1 and total STAT1 bands from infected samples were quantified as in **Figure 2B** and the amount of phosphorylated STAT1 was normalized to corresponding total STAT1 amount. The normalized amount of phosphorylated STAT1 in control siRNA samples was considered 100% for comparisons to HDAC2 siRNA samples. Data presented are means ± standard errors of the means of three independent experiments; asterisks indicate the significant differences in means. **(D,E)** A549 cells transfected with the control and HDAC2 siRNAs and infected with the WSN as above were harvested after 12 h of infection. **(D)** The HDAC2, viperin, actin and NP were detected in total uninfected and infected cell lysates by WB. **(E)** The viperin and actin bands from infected samples were quantified as in **Figure 2B** and the amount of viperin was normalized to corresponding actin amount. The normalized amount of viperin in control siRNA samples was considered 100% for comparisons to HDAC2 siRNA samples. Data presented are means ± standard errors of the means of three independent experiments; asterisks indicate the significant differences in means.

The data presented so far indicated that endogenous HDAC2 possesses anti-IAV properties. Next, we aimed to understand the antiviral mechanism of HDAC2. Hosts have evolved complex and sophisticated innate and adaptive mechanisms to counter virus infections. An antiviral state is created in the host cells as soon as they encounter an IAV particle and results in the secretion of type I IFNs (Garcia-Sastre, 2011). Secreted type I IFNs then engage the type I IFN receptor on host cells in an autocrine and paracrine manner and activate the cytoplasmic Janus kinases (JAKs), which, in turn, phosphorylate the cytoplasmic transcription factor Signal Transducer and Activator of Transcription I (STAT1). The phosphorylated STAT1 is then translocated to the nucleus, where it forms a transcription complex called as interferon-stimulated gene factor 3 (ISGF3). Subsequently, ISFG3 binds to interferon-stimulated response element (ISRE) of the ISGs in a sequence-specific manner and induces the expression of plethora (over 300) of ISGs. Many of these ISGs have been shown to inhibit IAV infection by targeting various steps of its life cycle (Garcia-Sastre, 2011). Previously, by mostly using the purified IFN-alpha or infection with viruses other than IAV, it has been demonstrated that class I and class II HDACs activity is required to create an antiviral state and activation of the type I IFN-mediated response and expression of ISGs (Nusinzon and Horvath, 2003; Chang et al., 2004; Sakamoto et al., 2004). However, a precise role of individual class I and class II HDACs in IFN response and subsequent expression of their effector proteins is yet to be elucidated. We recently demonstrated a critical role of class I and class II HDACs activity in the induction of IAV-mediated phosphorylation of STAT1 and expression of ISGs such as IFITM3, ISG15 and viperin (Nagesh and Husain, 2016). Furthermore, we also demonstrated that HDAC1 is important for the IAV-induced expression of viperin (Nagesh and Husain, 2016). Therefore, to get insight into the antiviral mechanism of HDAC2, we first investigated its role in the induction of IAV-mediated STAT1 phosphorylation and expression of viperin. To accomplish this, A549 cells transfected with the non-targeting control siRNA or HDAC2-targeting siRNA were infected with WSN at an MOI of 0.5. Cells were then harvested after 2, 6, 12, and 24 h of infection, processed, and levels of phosphorylated STAT1 (pSTAT1) and viperin were analyzed by WB. The phosphorylation of STAT1 was first detected in control siRNA-transfected cells within 2 h of infection and peaked at 12 h of infection, then subsided at 24 h of infection almost to the level of 2 h (**Figure 5A**); potentially due to the antagonism by IAV or caspase-mediated cleavage of total STAT1 (King and Goodbourn, 1998). However, the kinetics of the extent of STAT1 phosphorylation was slower in the absence of HDAC2. A noticeably decreased levels of pSTAT1 were detected in the cells transfected with HDAC2 siRNA compared to the cells transfected with control siRNA after 6 and 12 h of infection (**Figure 5A**). A parallel time-course experiment with UV-irradiated WSN inoculum was carried to rule out the presence of contaminating interferons in inoculum inducing the STAT1 phosphorylation (Supplementary Figure 5). On the other hand, the expression of viperin was first detected in control siRNA-transfected cells after 12 h of infection and peaked at 24 h of infection. However, similar to pSTAT1, the expression kinetics of viperin was slower in the absence of HDAC2. Viperin was barely detectable in HDAC2 siRNA-transfected cells after 12 h of infection, and after 24 h of infection, it was detected at a reduced level compared to the control siRNA-transfected cells (**Figure 5A**). To quantify the decrease in pSTAT1 and viperin levels in the absence of HDAC2, we repeated this experiment at least two more times and quantitated their levels at 12 h of infection. We found that there was a significant 41% (*P* < 0.01)) decrease in pSTAT1 level in HDAC2-depleted cells compared to the control cells (**Figures 5B,C**). Similarly, about 53% less (*P* = 0.001) viperin was detected in HDAC2-depleted cells than in the control cells (**Figures 5D,E**). These data indicated that HDAC2 is an important component of IAV-induced host innate antiviral response.

## Discussion

The experimental evidence presented here indicate that host HDAC2, like its ‘twin’ HDAC1 possesses anti-IAV properties and, in turn, IAV downregulates HDAC2 expression to undermine its antiviral effect. IAV downregulated HDAC2 expression at both mRNA and polypeptide level in H1N1 strain independent manner. The downregulation of HDAC2 mRNA by both PR8 and WSN strains was dose-dependent, but the magnitude of HDAC2 mRNA downregulation varied according to the reference genes used for normalization. When normalized to 18S RNA, a relatively stable reference gene, the downregulation of HDAC2 mRNA by WSN was more profound than by PR8. This could be attributed to no trypsin used for infecting the A549 cells with PR8. Trypsin was not used because A549 cell monolayers are sensitive to trypsin, but are susceptible to PR8 infection without it. However, under this condition, trypsin-independent WSN may grow faster in A549 cells than PR8. Nonetheless, when normalized to GAPDH and actin, other two commonly used reference genes, both WSN and PR8 had an almost identical effect on the downregulation of HDAC2 mRNA. Alternatively, IAV host shutoff mechanisms mediated by the NS1 or PA-X protein are potentially downregulating the HDAC2 mRNA level (Khaperskyy and McCormick, 2015), and the observed variations in mRNA downregulation by PR8 and WSN could be attributed to the strain-dependent host shutoff functions of NS1 and PA-X (Desmet et al., 2013; Khaperskyy et al., 2014). Nevertheless, we believe that IAV is dysregulating host HDAC2 mainly at the polypeptide level to undermine its antiviral function. Because, the reduction in HDAC2 polypeptide level in IAV-infected cells was, (a) H1N1 strain-independent and (b) sensitive to treatment with a proteasome inhibitor, hence almost identical to the dysregulation of HDAC1 by IAV (Nagesh and Husain, 2016). Furthermore, like HDAC1, HDAC2 also has been shown to undergo ubiquitination and consequently proteasomal degradation during virus infection as well as heterologous conditions (Kramer et al., 2003; Kwon et al., 2016; Romani et al., 2016). Therefore, it is highly plausible that IAV dysregulating the HDAC2 (alongside HDAC1) at the polypeptide level via proteasomal degradation. However, the ubiquitination of HDAC2 (and HDAC1) and identity of the ubiquitin ligases/conjugases that ubiquitinate them and facilitate their degradation in IAV-infected cells remains to be determined.

On its own, IAV knocks down the HDAC2 polypeptide expression by up to 47%. The additional knockdown of HDAC2 expression by up to 90% via RNA interference resulting into higher IAV growth characteristics (as determined by increase in virus titers and intracellular NP levels) indicated an anti-IAV role for host HDAC2. However, to confirm the antiviral function of HDAC2, it is important to analyze the IAV growth characteristics in HDAC2-overexpressing cells. Nonetheless, the 4.2-fold increase in IAV titers in HDAC2-depleted cells observed here was not profoundly different to the 3.2-fold increase in IAV titers observed in HDAC1-depleted cells under similar conditions (Nagesh and Husain, 2016). This prompted two obvious questions, (1) whether the loss of HDAC2 is being compensated by the HDAC1, because it has been shown earlier in the heterologous systems that HDAC1 expression goes up when HDAC2 expression is ablated and vice versa (Jurkin et al., 2011; Kobayashi et al., 2017), and (2) whether simultaneous knockdown of HDAC2 and HDAC1 expression would have an additive effect on the increase in IAV virus titers. As of now, the answer to first question is potentially no, because we have observed that IAV independently downregulates the expression of both HDAC1 (Nagesh and Husain, 2016) and HDAC2 (reported here). Furthermore, as reported elsewhere (Jurkin et al., 2011; Kobayashi et al., 2017), we confirmed the upregulation of HDAC1 polypeptide level in HDAC2-depleted A549 cells in the absence of IAV infection (Supplementary Figure 6, lane 2). However, such upregulated level of HDAC1 polypeptide also was subsequently downregulated upon IAV infection (Supplementary Figure 6, lane 4). This indicated that IAV is simultaneously undermining the compensatory role of HDAC1 while dysregulating the HDAC2, and possibly vice versa. Moreover, the upregulated expression levels of HDAC2 or HDAC1 in the absence of the other do not always compensate for their function (Lagger et al., 2002; Dovey et al., 2010; Hagelkruys et al., 2015; Kobayashi et al., 2017). The answer to second question is also possibly no, because in a preliminary experiment we did not observe an additive effect of HDAC2/1 double knockdown on the increase in IAV titers compared to the single knockdown of either HDAC2 or HDAC1 (data not shown). One of the possible explanations for this could be an additive effect of HDAC2/1 double knockdown on the reduced cell viability as observed elsewhere (Ler et al., 2015). Nonetheless, future investigations systematically examining the effect of combined depletion of HDAC2/1 as well as the individual role of HDAC3 (another closely related class I member; Yang et al., 2002) alone or in conjunction with HDAC2 and HDAC1 in IAV infection would potentially provide definitive answers to the above and related questions.

A significant decrease in the phosphorylation of STAT1 and expression of viperin in HDAC2-depleted infected cells indicated a positive involvement of HDAC2 in the IAV-induced host innate antiviral response. This was consistent with our previous observation wherein an inhibitor of class I and class II HDACs, trichostatin A significantly reduced the IAV-induced phosphorylation of STAT1 and expression of viperin (Nagesh and Husain, 2016). Furthermore, the reduction in IAV-induced viperin expression in HDAC2-depleted cells was also analogous to the reduction in IAV-induced viperin expression in HDAC1-depleted cells (Nagesh and Husain, 2016). Therefore, it is likely that HDAC2 forms a complex with HDAC1 that co-activates the expression of viperin in IAV-infected cells, and this step potentially represents the redundant function of HDAC2 and HDAC1 during IAV infection. Previously, HDAC2 and HDAC1 have been shown to form a complex with promyelocytic leukemia (PML) protein that co-activates the expression of another ISG, ISG54 in human cytomegalovirus-infected cells (Kim and Ahn, 2015). Furthermore, HDAC1 has also been shown to form a complex with promyelocytic leukemia zinc finger (PLZF) protein to co-activate the viperin expression induced by IFN-alpha (Xu et al., 2009). On the other hand, the involvement of HDAC2 in IAV-induced phosphorylation of STAT1 seems to be independent of HDAC1. Unlike in HDAC2-depleted cells, the IAV-induced phosphorylation of STAT1 remained largely unchanged in HDAC1-depleted cells (Nagesh and Husain, unpublished). Therefore, this step potentially represents the non-redundant or partially redundant function of HDAC2 with respect to HDAC1 in IAV-induced host innate antiviral response. This would not be unprecedented as HDAC2 and HDAC1 have been shown to exhibit non-redundant or partially redundant functions in heterologous systems (Dovey et al., 2010; Hagelkruys et al., 2015). Evidently, both IFN-alpha and IFN-gamma induce the phosphorylation of STAT1 during innate immune response. However, trichostatin A, an inhibitor of both class I and class II HDACs, only inhibited the STAT1 phosphorylation induced by exogenously added IFN-gamma (Klampfer et al., 2004), not IFN-alpha (Nusinzon and Horvath, 2003; Chang et al., 2004). Similarly, in HDAC2-depleted cells, the IFN-gamma-induced activation of STAT1 was also downregulated (Klampfer et al., 2004). In addition to type I (IFN-alpha) and type III IFN, IAV also induces the IFN-gamma (type II IFN) response in infected cells, including A549 (Sun and Metzger, 2008; Uetani et al., 2008). Thereby, we previously observed a markedly reduced phosphorylation of STAT1 in IAV-infected cells treated with trichostatin A (which also inhibits HDAC2 activity), but not in HDAC1-depleted IAV-infected cells (Nagesh and Husain, 2016). In contrast, herein, we observed a significantly reduced phosphorylation of STAT1 in HDAC2-depleted IAV-infected cells. This indicated that HDAC2 potentially regulates the IAV-induced STAT1 phosphorylation in response to IFN-gamma, and independent of HDAC1. One possible mechanism for HDAC2 to positively regulate such IAV-induced STAT1 phosphorylation is through the acetylation-phosphorylation switch (Kramer et al., 2009). In addition to phosphorylation, STAT1 also known to undergo acetylation, which negatively regulates its phosphorylation and consequently its activation. The HDAC3, another class I HDAC, has been shown to reverse this phenomenon by deacetylating the STAT1 and facilitating its phosphorylation (Kramer et al., 2009). A similar phenomenon might be occurring in IAV-infected cells vis-a-vis HDAC2; hence, IAV downregulates the HDAC2 polypeptide expression to potentially prevent the deacetylation of STAT1 and consequently its phosphorylation and activation. However, this remains to be experimentally validated. Furthermore, it is unlikely that viperin is the only ISG, the expression of which HDAC2 is involved in. A direct involvement of HDAC2 in the expression of ISGs that inhibit IAV infection remains to be investigated.

In summary, we provide the evidence for another classical HDAC to possess anti-IAV properties and an insight into its antiviral mechanism. The classical HDACs are seem to be another integral component of IAV-induced host innate antiviral response, and elucidating their precise roles in the intricate innate immune pathways as well as co-operative or compensatory behavior is looking to be an exciting journey ahead. The IAV remains to be one of the most significant human respiratory pathogens, and constant emergence of novel and drug-resistant IAV variants and lack of a universal vaccine continuously make the case for developing the novel alternative anti-IAV strategies. Because there is a considerable interest in targeting the HDACs to treat various human diseases, a clear molecular understanding of IAV-HDACs interplay may lead to the development of one such a strategy.

## Author Contributions

MH conceived the study. MH and PN designed the study, and PN, MzH, and HG carried out the experiments. PN and MH interpreted the results and wrote the manuscript.

## Conflict of Interest Statement

The authors declare that the research was conducted in the absence of any commercial or financial relationships that could be construed as a potential conflict of interest.
