# Supplementary material for: Histone Deacetylase 2 Is a Component of Influenza A Virus-Induced Host Antiviral Response
Source: Front Microbiol. 2017 Jul 17;8:1315. doi: 10.3389/fmicb.2017.01315 (PMC5511851; doi:10.3389/fmicb.2017.01315)
Supplement: Supplementary file 1 [file Image_1.PDF]

Supplementary Material

Histone deacetylase 2 is a component of influenza A virus-induced host antiviral response

Prashanth Thevkar Nagesh, Mazhar Hussain, Henry David Galvin and Matloob Husain*

*** Correspondence:** Dr. Matloob Husain, matloob.husain@otago.ac.nz

Supplementary Figures

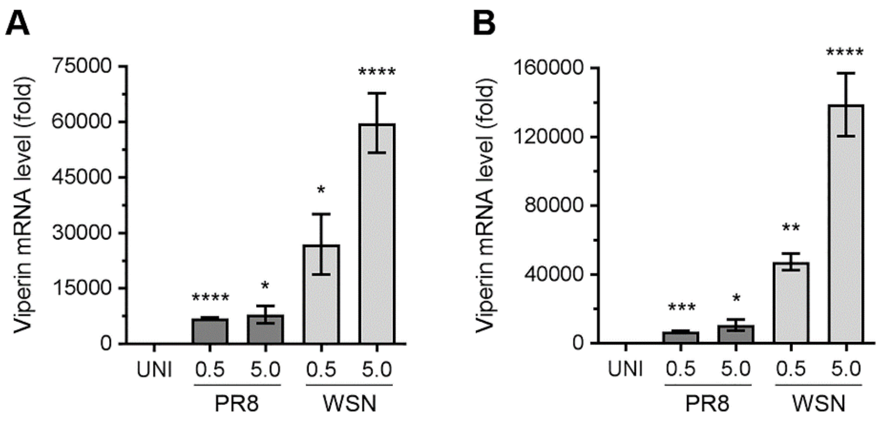

Supplementary Figure 1. A549 cells were infected with PR8 or WSN at an MOI of 0.5 and 5.0 for 24 h. The uninfected (UNI) and infected (0.5, 5.0) cells were harvested, processed, and viperin mRNA was detected by quantitative real-time PCR. Alongside, the GAPDH and actin mRNAs were detected as a reference and used to normalize the viperin mRNA levels shown in panel A and B, respectively. The normalized value of viperin mRNA in UNI sample was considered 1-fold for comparison to infected sample. Error bar represents means ± standard errors of the means of three independent experiments; asterisks indicate the significant differences in means.

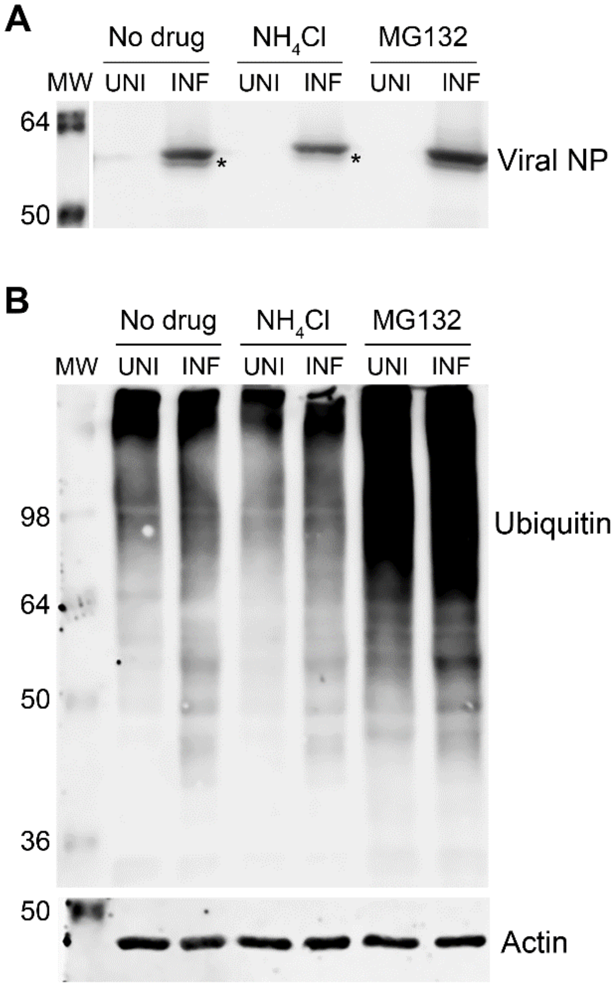

Supplementary Figure 2. A549 cells were infected with WSN at an MOI of 0.5, and subsequently treated with NH_4Cl (20 mM) or MG132 (10 µM) for 24 h. (A) Viral NP, and (B) ubiquitin and actin were detected in total uninfected (UNI) and infected (INF) cell lysates by WB. MW, molecular weights.

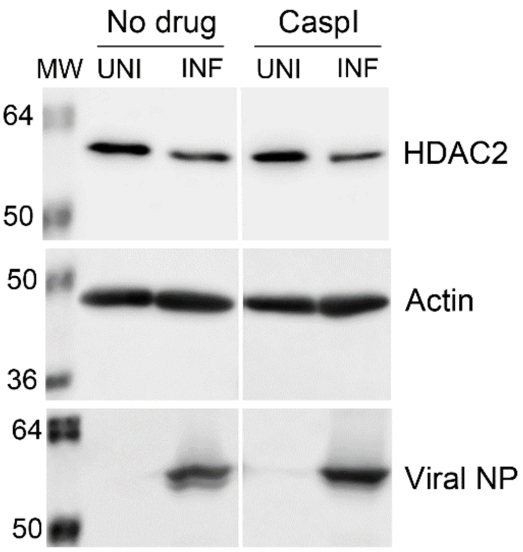

Supplementary Figure 3. A549 cells were infected with WSN at an MOI of 0.5, and subsequently treated with caspase 3 inhibitor (40 µM) for 24 h. Total cell lysates were prepared, and HDAC2, actin and viral NP were detected in uninfected (UNI) and infected (INF) cell lysates by WB. CaspI, caspase 3 inhibitor; MW, molecular weights. *Note: reduced level of NP cleavage product in lane 4 indicate the potency of caspase 3 inhibitor.*

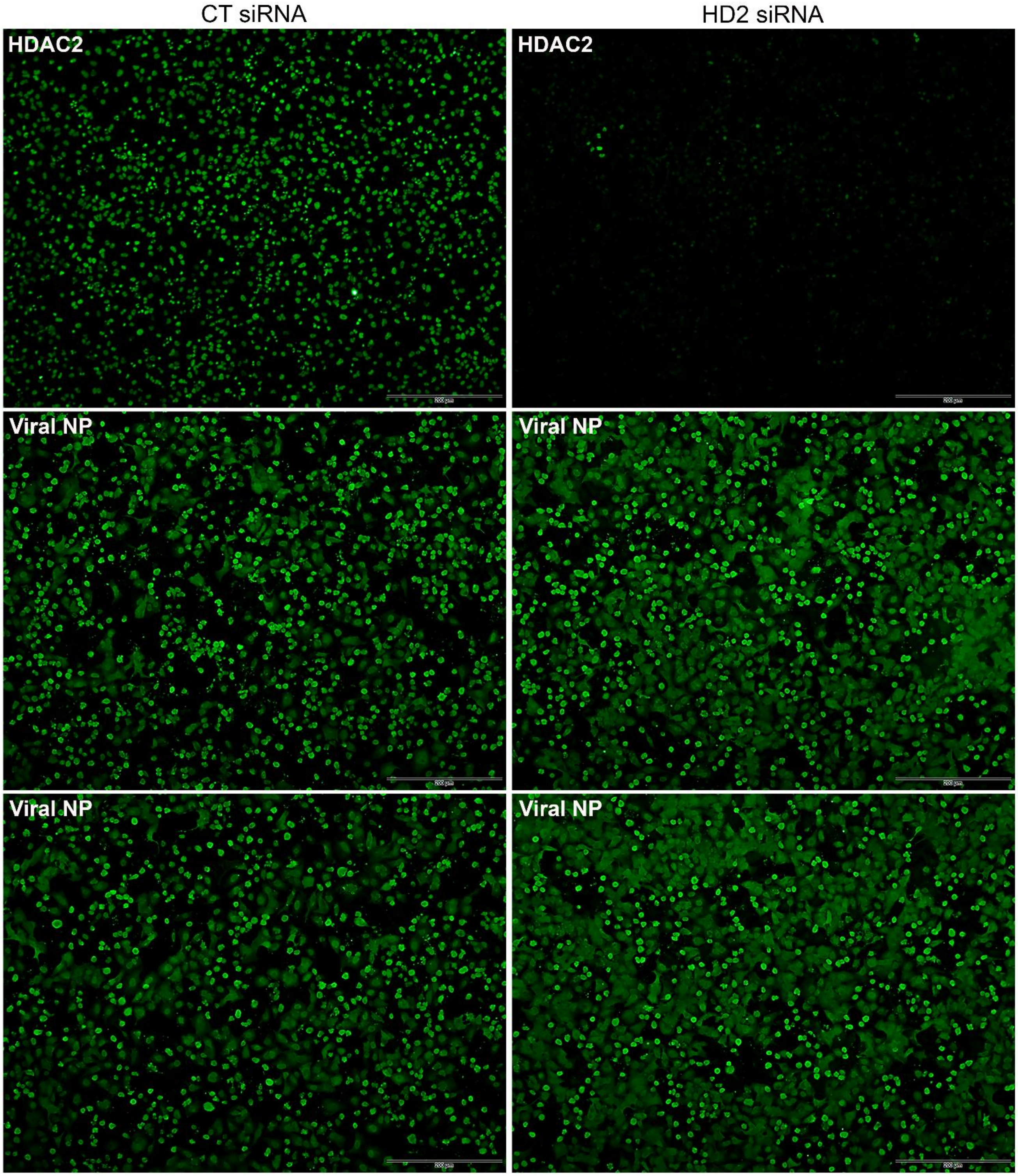

Supplementary Figure 4. A549 cells transfected with the control (CT) siRNA or HDAC2 (HD2) siRNA for 72 h were infected with WSN at an MOI of 0.5. After 24 h, cells were stained with mouse anti-HDAC2 (1:500, first row) or mouse anti-NP (1:500, second and third row) antibody followed by Alexa 488-conjugated anti-mouse IgG antibody (1:500; Life Technologies) and DNA-binding dye Hoechst (10 µg/ml; Life Technologies) and visualized under the same conditions as described

elsewhere (Husain and Cheung, 2014). *One image of HDAC2 staining and two images of viral NP staining (above) with their corresponding nuclei staining (below) from each sample are shown.* Scale bar, 200 µm

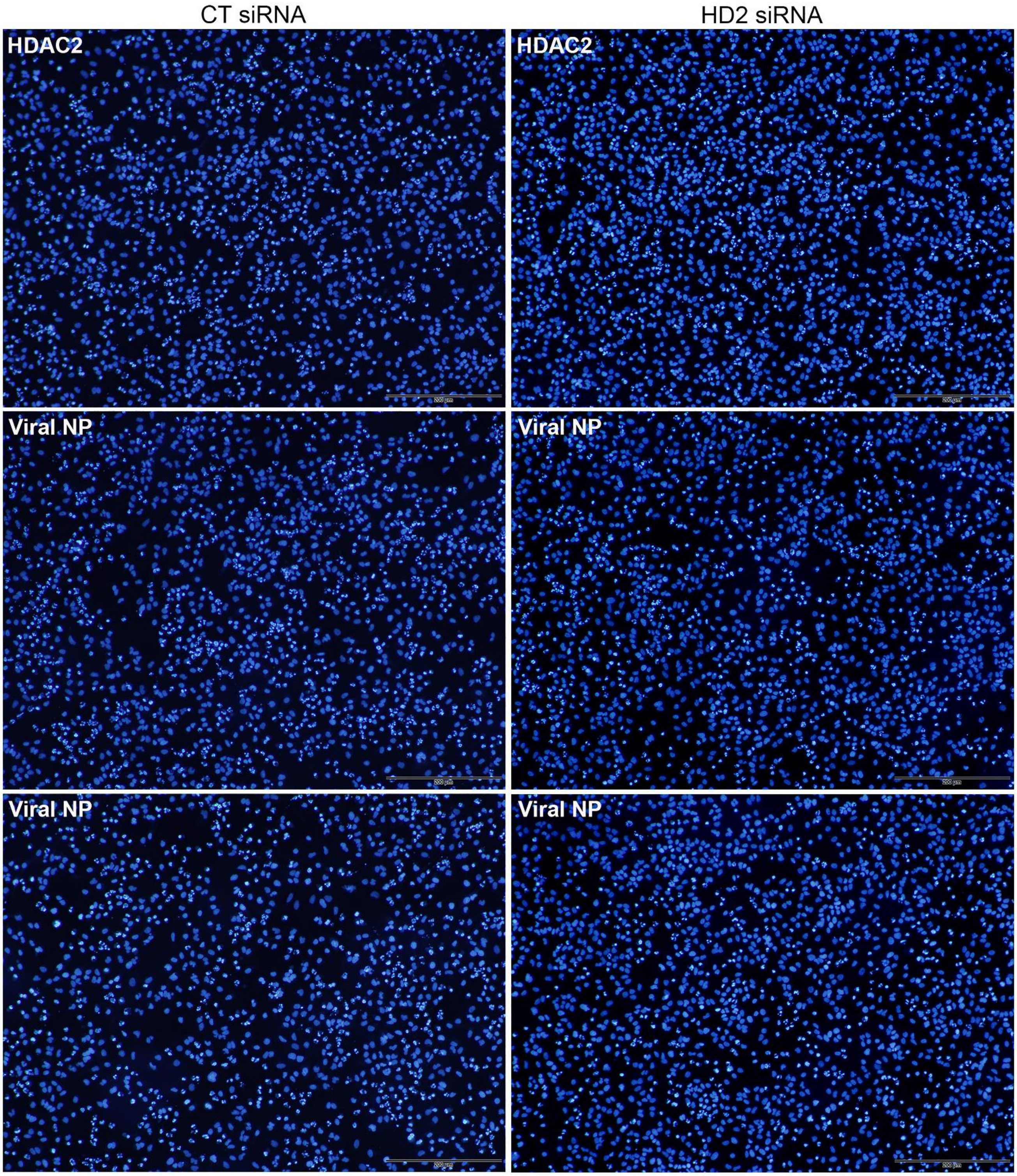

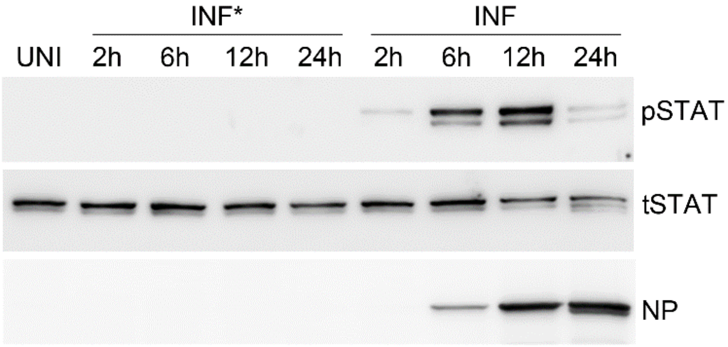

Supplementary Figure 5. A549 cells were infected with either UV-irradiated (INF*) or live (INF) WSN at an MOI of 0.5. Cells were harvested at the indicated times and total cell lysates were prepared, and phosphorylated STAT1 (pSTAT), total STAT1 (tSTAT), and viral NP were detected in uninfected (UNI) and infected (2h, 6h, 12h, 24h) cell lysates by WB.

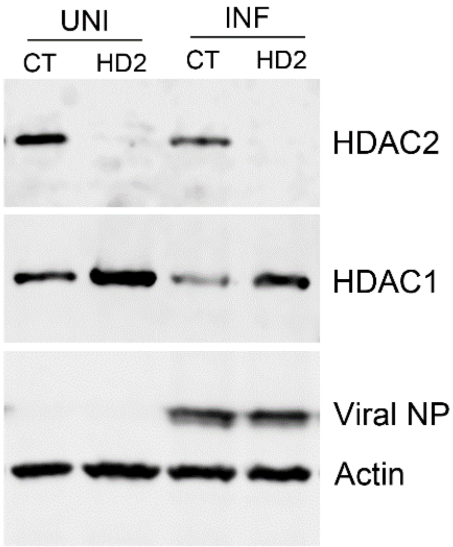

Supplementary Figure 6. A549 cells were transfected with control (CT) siRNA or HDAC2 (HD2) siRNA for 72 h, and subsequently infected with WSN at an MOI of 0.5 for 24 h. Total cell lysates were prepared, and HDAC2, HDAC1, viral NP and actin were detected in uninfected (UNI) and infected (INF) cell lysates by WB.
